# Karyotype diversity and evolutionary trends in the Asian swamp eel *Monopterus albus* (Synbranchiformes, Synbranchidae): a case of chromosomal speciation?

**DOI:** 10.1186/s12862-019-1393-4

**Published:** 2019-03-08

**Authors:** Weerayuth Supiwong, Krit Pinthong, Kriengkrai Seetapan, Pasakorn Saenjundaeng, Luiz A. C. Bertollo, Ezequiel A. de Oliveira, Cassia F. Yano, Thomas Liehr, Sumalee Phimphan, Alongklod Tanomtong, Marcelo B Cioffi

**Affiliations:** 1 Faculty of Applied Science and Engineering, Khon Kaen University, Nong Khai Campus, Muang, Nong Khai, 34000 Thailand; 2 Department of Fundamental Science, Faculty of Science and Technology, Surindra Rajabhat University, Muang, Surin, 32000 Thailand; 3 School of Agriculture and Natural Resources, University of Phayao, Tumbol Maeka, Muang, Phayao, 56000 Thailand; 4 Departamento de Genética e Evolução, Universidade Federal de São Carlos, São Carlos, São Paulo Brazil; 5 Jena University Hospital, Friedrich Schiller University, Institute of Human Genetics, Kollegiengasse 10, D-07743 Jena, Germany; 6 Toxic Substances in Livestock and Aquatic Animals Research Group, Khon Kaen University, Muang, Khon Kaen, 40002 Thailand

**Keywords:** Tropical freshwater fish, Reproductive isolation, Repetitive DNAs, Centric fusion, Species complex

## Abstract

**Background:**

Synbranchidae or swamp eels are fishes belonging to the order Synbranchiformes that occur in both freshwater and occasionally in brackish. They are worldwide distributed in tropical and subtropical rivers of four different continents. A large degree of chromosomal variation has been found in this family, mainly through the use of conventional cytogenetic investigations. Inside this group, a still almost unexplored species under the cytogenetic point of view is the Asian swamp eel *Monopterus albus*, a widely distributed species throughout Asia. Here, we tested the hypothesis of chromosomal speciation, where a case of sympatric speciation may occur as the primary consequence of chromosomal rearrangements. We performed a comparative chromosomal analysis of *M. albus* from 22 different localities in Thailand, using distinct staining methods (C-banding, Ag-NO_3,_ and Chromomycin A_3_), and FISH with repetitive DNA probes (5S rDNA, 18S rDNA, *Rex1* element and microsatellite repeats).

**Results:**

This approach evidenced two contrasting karyotypes (named karyomorphs A and B) that varied concerning their 2n and repetitive DNAs distribution, where chromosomal fusions and pericentric inversions were involved in such differentiation. While the karyomorph A has 2n = 24 chromosomes, the karyomorph B has only 2n = 18, both with NF = 24. In addition, karyomorph A contains only acrocentric chromosomes, while karyomorph B contains three unique metacentric pairs. These features highlight that *M. albus* has already gone through a significant genomic divergence, and may include at least two cryptic species.

**Conclusions:**

This marked chromosomal differentiation, likely linked to the lifestyle of these fishes, point to the occurrence of a chromosomal speciation scenario, in which fusions and inversions had a prominent role. This highlights the biodiversity of *M. albus* and justifies its taxonomic revision, since this nominal species may constitute a species complex.

## Background

Freshwater habitats make up less than 0.01% of available aquatic habitat but contain almost half of all 34,000 valid fish species, making freshwater fishes an excellent model for studying speciation events [1, 2]. However, although freshwater environments are largely fragmented and isolated, which means allopatric speciation events are more frequently found, several known cases of sympatric speciation have already been identified [3, 4].

In recent years, cytogenetic studies have made important contributions toward a better understanding of recent speciation events, since chromosomal rearrangements can act as genetic barriers to gene flow, thus facilitating reproductive isolation [5–8]. The chromosomal rearrangements promote the reorganization of the genetic structure, and the evolutionary impact and the consequences at the speciation level can vary according to the rearrangement type, that is, inversion, fusion, fission or translocation [7–12]. Such chromosomal rearrangements can facilitate adaptation to heterogeneous environments by limiting genomic recombination [10].

Molecular cytogenetic studies using fluorescence in situ hybridization (FISH) to map repetitive DNA sequences have provided important contributions to the characterization of chromosomal rearrangements and the evolution of distinct fish groups (reviewed in [13]). Repetitive DNAs, widely distributed in the eukaryotic genomes, are generally divided into two classes, one comprising tandem sequences (satellite DNAs, minisatellites and microsatellites), and the other comprising interspersed sequences, such as transposons and retrotransposons [14].

**Fig. 1:**
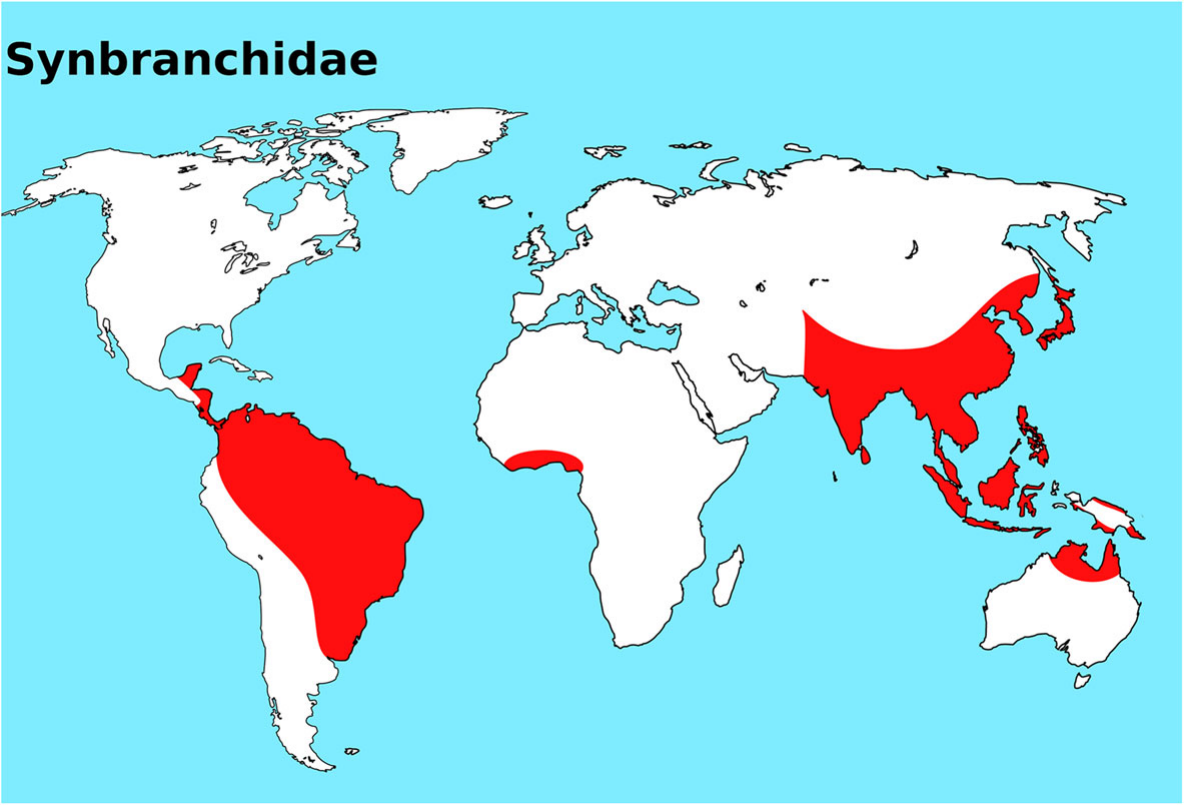
World map highlighting the actual distribution of Synbranchidae fish family. This map was created using the following softwares: QGis 3.4.3 and Inkscape 0.92

Synbranchidae or swamp eels are fishes belonging to the order Synbranchiformes; they occur in freshwater and occasionally in brackish water. They are distributed worldwide in tropical and subtropical Asia, the Indo-Australian Archipelago, West Africa (Liberia), Mexico and Central and South America (Fig. 1) [1]. This family comprises four genera: *Macrotrema, Monopterus*, *Ophisternon* and *Synbranchus* [15]. While *Macrotrema, Monopterus* and *Ophisternon* are found in the Old World, *Ophisternon* and *Synbranchus* occur in the New World. Thus, the genus *Ophisternon* is currently found in both the Old and New Worlds. *Macrotrema* is restricted to Asia and *Synbranchus* to the Neotropical region. At present, 23 valid species are recognized: *Macrotrema* (1); *Monopterus* (13*)*, *Ophisternon* (6*)* and *Synbranchus* (3) [16]. *Macrotrema caligans* (Cantor, 1849), *Monopterus albus* (Zuiew, 1793) and *Ophisternon bengalense* (McClelland, 1844) are the three species recorded in Thailand [1].

*Monopterus albus*, commonly known as the Asian swamp eel or rice field eel, is widely distributed throughout Asia, from northern India and Burma to China, Asiatic Russia, Japan and the Indo-Malayan Archipelago. However, cytogenetic studies conducted on this species are scarce. There are only three karyotype reports for *M. albus* from Thailand and China, showing 24 chromosomes and the fundamental number NF = 24 [17–19]. However, Donsakul and Magtoon [17] have also reported that *M. albus* from the central part of Thailand differs from the other populations, as it has 2n = 18 and NF = 24 (Table 1). Similarly, the Venezuelan *Ophisternon aenigmaticum* also highlights a karyotype diversification, including 2n = 45 and NF = 51 and 2n = 46 and NF = 52 [20], as well as the *Synbranchus marmoratus* species from Brazil and Argentina, which presents 2n ranging from 42 to 46 [21–25]. The Synbranchidae family has high karyotype diversity among its species, with 2n ranging from 18 to 46, mainly due to extensive chromosomal rearrangements (fusions/fissions and inversions) (Table 1).

**Table 1:** Comparative cytogenetic data of Symbranchidae species

Species	Site sampling	2n	NF	Karyotype	Reference
*Monopterus albus*	Central of Thailand	18	24	6 m + 12a	[6]
	Northeast of Thailand	24	24	24a	[6]
	Central of Thailand	24	24	24a	[8]
	China	24	24	24a	[7]
*M. cuchia*	India	42	46	4sm + 38a	[33]
*Ophisternon aenigmaticum*	La Vega, Garcia, Venezuela	45	51	6 m + 39a	[9]
	EL Valle, Garcia, Venezuela	46	52	6 m + 40a	
*O. bangalense*	Southeast coast of India	46		42 m + 4sm	[34]
*Synbranchus lampreia*	Lago Catalão	44	50	6 m + 2st + 36a	[19]
*S. madeirae*	Lago Catalão	46	52	6 m + 2st + 38a	
*S. marmoratus*	Bataguassu – MS, Igaraçu do Tietê – SP, Pirassununga – SP, Icém – SP, Cáceres – MT, BR	42	46	4 m + 12st + 26a	[14]
*Synbranchus marmoratus*	Coxim, MS, BR	42	46	4 m,sm + 38st,a	[10]
	São Simão, GO, BR	42	46	4 m,sm + 38st,a	
	Nova Granada, SP, BR	42	46	4 m,sm + 38st,a	
	Botucatu, SP, BR	42	46	4 m,sm + 38st,a	[11]
	Birigui, SP, BR	42	46	4 m,sm + 38st,a	
	ParaguaçuPaulista, SP, BR	42	46	4 m,sm + 38st,a	
	Pirassununga, SP, BR	42	48	6 m,sm + 36st,a	
	RibeirãoPreto, SP, BR	42	48	6 m,sm + 36st,a	
	Bataguaçu, MS, BR	42	48	6 m,sm + 36st,a	
	Guaíra – PR, BR	42	48	6 m + 10st + 26a	[14]
	Londrina, PR, BR	42	52	4 m + 2sm + 8st + 28a	[13]
	Guairá, PR, BR	42	52	4 m + 2sm + 8st + 28a	
	Miranda, MS, BR	42	52	4 m + 2sm + 8st + 28a	
	Pereiras, SP, BR	42	52	4 m + 6sm + 8st + 24a	
	PresidenteEpitácio, SP, BR	42	52	4 m + 6sm + 8st + 24a	
	Rio Claro, SP, BR	44	48	4 m,sm + 40st,a	[10]
	Pentecostes, CE, BR	44	48	4 m,sm + 40st,a	
	Botucatu, SP, BR	44	48	4 m,sm + 40st,a	[11]
	Birigui, SP, BR	44	48	4 m,sm + 40st,a	
	Bataguaçu, MS, BR	44	48	4 m,sm + 40st,a	
	Ituzaingó, Corrientes, AR	44	48	4 m,sm + 40st,a	[12]
	Reconquista, Santa Fé, AR	44	48	4 m,sm + 40st,a	
	Garabato, Santa Fé, AR	44	48	4 m,sm + 40st,a	
	Cerrito – RS, Rio Branco – AC, BR	44	48	4 m + 10st + 30a	[14]
	Pirassununga, SP, BR	44	50	4 m + 2sm + 8st + 30a	[13]
	Pirassununga, SP, BR	46	50	4 m,sm + 42st,a	[11]
	RibeirãoPreto, SP, BR	46	50	4 m,sm + 42st,a	
	Bandeirantes, PR, BR	46	52	4 m + 2sm + 8st + 32a	[13]
	Igaraçu do Tietê – SP, Pirassununga – SP, BR	46	52	4 m + 10st + 32a	[14]
	Pirassununga – SP, Icém – SP, BR	46	52	6 m + 10st + 30a	
	Miranda, MS, BR	46	54	6 m + 2sm + 6st + 32a	[13]

Notes: *BR* Brazil, *AR* Argentina, *2n* diploid number, *NF* fundamental number, *m* metacentric, *sm* submetacentric, *st* subtelocentric and *a* acrocentric chromosomes

**Fig. 2:**
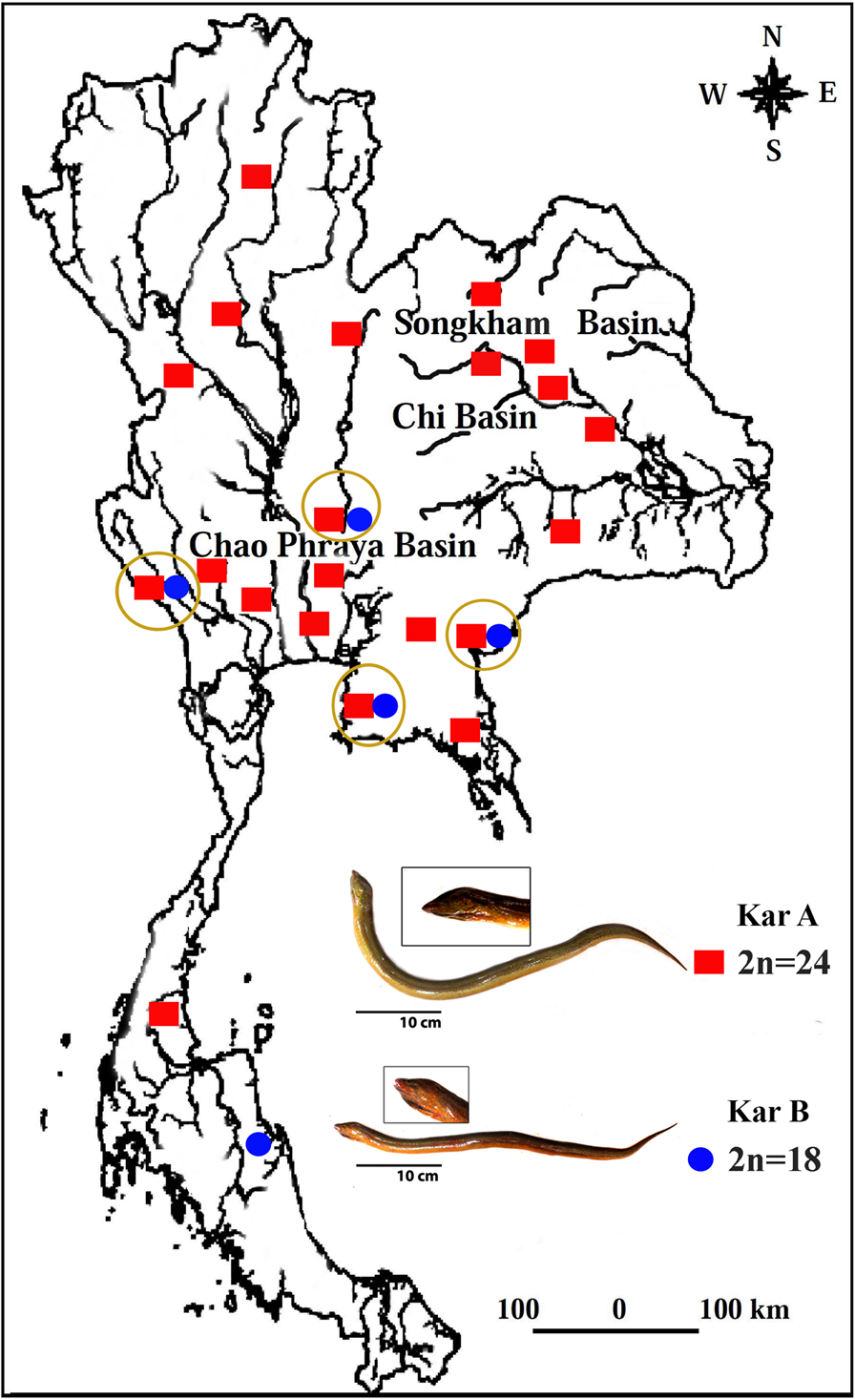
Map of Thailand highlighting the sample sites and specimens of two karyomorphs of the Asian swamp eel *Monopterus albus*. Red squares = karyomorph A (2n = 24) and blue circles = karyomorph B (2n = 18). Sympatry of both karyomorphs is indicated by circles. This map was created using the following software: Adobe Photoshop 7.0

This study presents a comparative chromosomal analysis of *Monopterus albus* from 22 different localities in Thailand (Fig. 2), using distinct staining methods (C-banding, Ag-NO_3,_ and chromomycin A_3_) as well as FISH with repetitive DNA probes (5S rDNA, 18S rDNA, *Rex1* element and microsatellite repeats). We tested the hypothesis of chromosomal speciation, where a case of sympatric speciation may occur as the primary consequence of chromosomal rearrangements. This approach provided an in-depth karyotype characterization of this taxon, evidencing the presence of two contrasting karyotypes occurring in sympatry and the occurrence of likely distinct species in this nominal species. This marked chromosomal differentiation, likely linked to the lifestyle of these fishes and their population fragmentation, protects from gene flow and therefore promotes speciation.

## Results

### Karyotypes

**Fig. 3:**
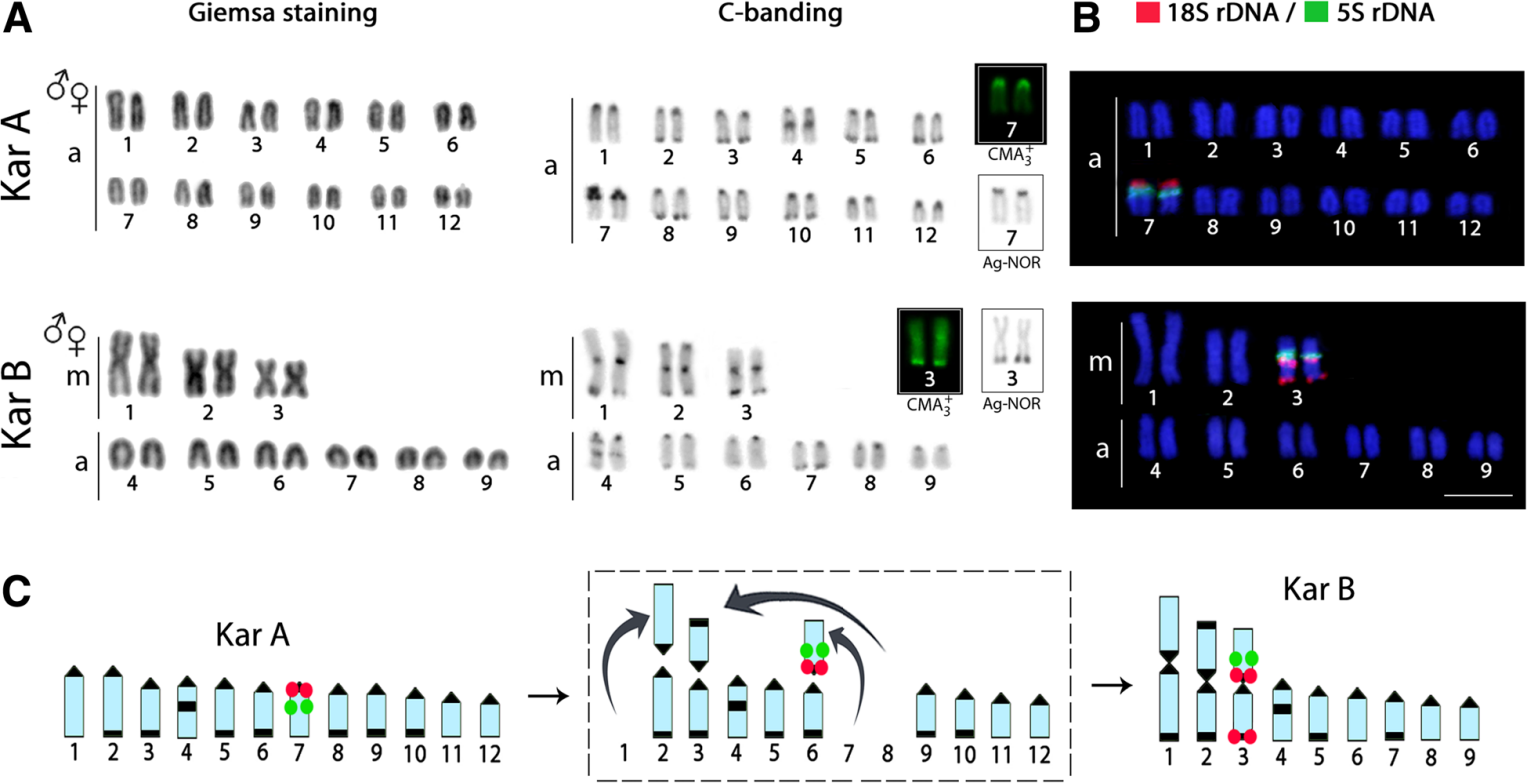
Karyotypes of the two Asian swamp eel *Monopterus albus* karyomorphs analyzed by (**a**) conventional staining and C-banding and (**b**) fluorescence in situ hybridization (FISH) with 5S (green) and 18S (red) rDNA probes. Inserted boxes indicate NOR-bearing chromosomes and CMA3 positive chromosomes. In (**c**) idiograms highlight the distribution of 18S rDNA (red) and 5S rDNA (green) in both karyomorphs. In the middle, the panel label highlights the main rearrangements related to karyotype differentiations, where centric fusions have played a major role. Scale bar = 5 μm

*M. albus* from the populations analyzed presented two distinct karyotype forms, one with 2n = 24 (24a) and NF = 24 (karyomorph A) and the other with 2n = 18 (6 m + 12a) and NF = 24 (karyomorph B) (Fig. 3a). The first karyomorph was present in 21 localities (183 specimens), while the latter was found in five (17 specimens). Sympatry of both karyomorphs was observed in four localities, namely Nakhon Nayok, Kanchanaburi (Sri Yok), Chon Buri and Sa Kaeo Provinces (Table 2 and Fig. 2). No heterozygous karyotype forms were observed in the four localities of sympatry.

**Table 2:** Chromosomal data for *Monopterus albus* populations from different Thailand regions

Region	Population/Province	Number of specimen	2n	NF	Karyotype	NORs
Central	Nakhon Nayok	5	18	24	6 m + 12a	2 (near telomere)
		7	24	24	24a	2 (near cetromere)
	Nakhon Pathom	15	24	24	24a	2 (near cetromere)
	Bangkok	8	24	24	24a	2 (near cetromere)
	Sing Buri	10	24	24	24a	2 (near cetromere)
South	Nakhon Si Thammarat	8	18	24	6 m + 12a	2 (near telomere)
	Chumphon	8	24	24	24a	2 (near cetromere)
West	Kanchanaburi	6	18	24	6 m + 12a	2 (near telomere)
	(Sri Yok)	8	24	24	24a	2 (near cetromere)
	Kanchanaburi (Sri Sawat)	11	24	24	24a	2 (near cetromere)
East	Chon Buri	6	18	24	6 m + 12a	2 (near telomere)
		10	24	24	24a	2 (near cetromere)
	Sa Kaeo (including	9	18	24	6 m + 12a	2 (near telomere)
	Campodia)	6	24	24	24a	2 (near cetromere)
	Chanthaburi	15	24	24	24a	2 (near cetromere)
	PrachinBuri	12	24	24	24a	2 (near cetromere)
North	Tak	8	24	24	24a	2 (near cetromere)
	Phayao	10	24	24	24a	2 (near cetromere)
	Sukhothai	10	24	24	24a	2 (near cetromere)
Northeast	KhonKaen	12	24	24	24a	2 (near cetromere)
	Loei	8	24	24	24a	2 (near cetromere)
	Roi Et	8	24	24	24a	2 (near cetromere)
	Kalasin	5	24	24	24a	2 (near cetromere)
	Buri Ram	6	24	24	24a	2 (near cetromere)
	UdonThani	8	24	24	24a	2 (near cetromere)
	Mahasarakham	4	24	24	24a	2 (near cetromere)

Notes: *2n* diploid number, *NF* fundamental number, *m* metacentric and *a* acrocentric chromosomes

### C-banding, ag-NORs and Chromomycin A3 staining

C-positive heterochromatic bands were observed in the centromeric/pericentromeric region of all chromosomes as well as in the telomeric region of several pairs in both karyomorphs**.** Interstitial heterochromatic blocks were also found in chromosome pair no. 4 in both karyomorphs *(*Fig. 3a*)*. Ag-NORs sites were present in the centromeric region of pair no. 7 and on the telomeric region of pair no. 3 in karyomorphs A and B, respectively. These Ag-NORs were the only observed GC-rich regions in the karyotype *(*Fig. 3a, boxed*)*.

### Chromosome mapping of 5S and 18S rDNAs

5S rDNA sequences were found in the pericentromeric region of the q arms of chromosome pair no. 7 in karyomorph A and in the pericentromeric region of the p arms of chromosome pair no. 3 in karyomorph B (Fig. 3b). Concerning the 18S rDNA sequences, chromosome pair no. 7 of karyomorph A also displayed sites in the pericentromeric region of the q arms, while in karyomorph B, in addition to the signals in the pericentromeric region, chromosomal pair no. 3 also showed telomeric markings (Fig. 3b). Therefore, the 5S and 18S rDNAs are located together in one chromosome pair in both karyomorphs.

### Microsatellites and Rex1 distribution

**Fig. 4:**
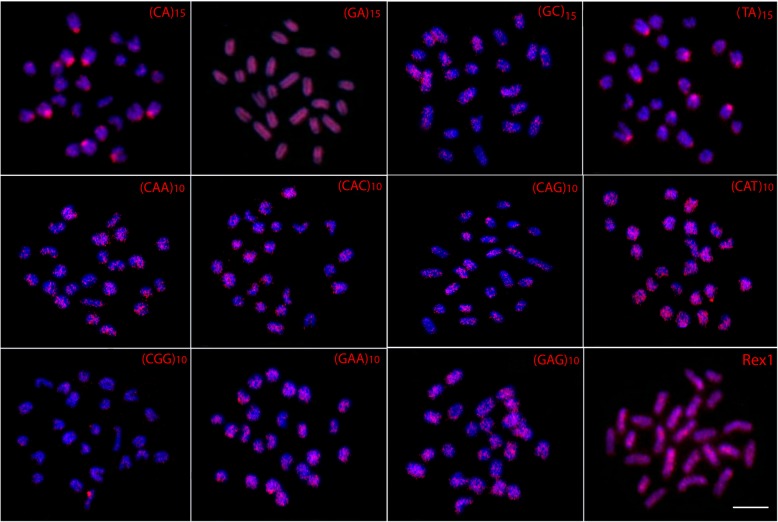
Metaphase plates of *Monopterus albus* karyomorph A mapped with di- and trinucleotide microsatellites and Rex1 as probes. Scale bar = 5 μm

**Fig. 5:**
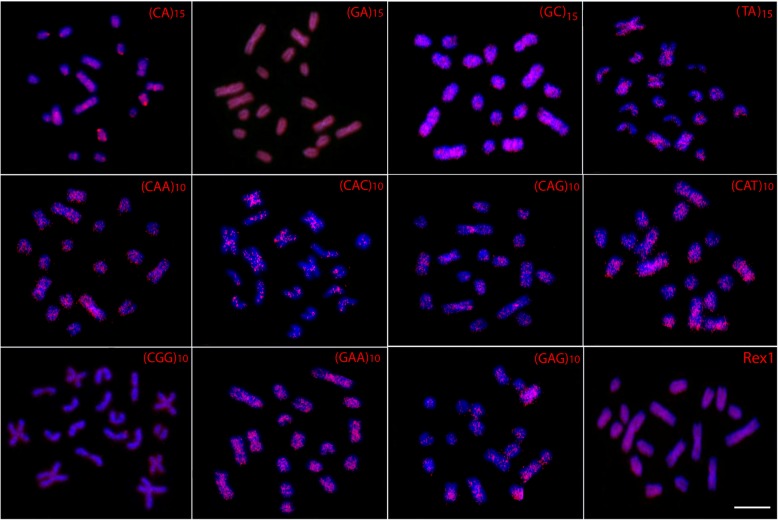
Metaphase plates *Monopterus albus* karyomorph B mapped with di- and trinucleotide microsatellites and *Rex1* as probes. Scale bar =5 μm

The (GC)_15_, (CAA)_10_, (CAC)_10_, (CAG)_10_, (CAT)_10_, (CGG)_10_, (GAA)_10_ and (GAG)_10_ repeats displayed scattered hybridization signals throughout the genome of both karyomorphs, some showing more concentrated signals. However, while the (CA)_15_ and (TA)_15_ sequences also show a scattered distribution in karyomorph B, they clearly accumulate at the centromeric region of several chromosomes in karyomorph A. In turn, the (GA)_15_ and the retroelement *Rex1* sequences present a strong, dispersed distribution without preferential accumulation in any chromosome pairs of either karyomorph (Figs. 4 and 5).

## Discussion

The occurrence of distinct *M. albus* karyomorphs living in sympatry with the absence of natural hybrids, as found in the present study in Nakhon Nayok, Kanchanaburi (Sri Yok), Chon Buri and Sa Kaeo Provinces, reinforces the hypothesis that these karyomorphs represent two reproductively isolated biological units. Although easily distinguishable through cytogenetic analysis, specimens from both karyomorphs have the same morphology, making difficult the identification of such probable new species.

The integration of both conventional and molecular cytogenetic approaches allowed the proposal of some chromosomal rearrangements probably related to the differentiation of both *M. albus* karyomorphs**,** where centric fusions appear as the main evolutionary sources shaping such a process (Fig. 3c). The different karyotype composition among individuals allowed us to identify two distinct karyomorphs, named A and B, which presented 2n of 24 and 18 chromosomes, respectively, both with NF = 24. The chromosomal divergence as well as the relation between the karyomorphs is clearly evidenced by their same NF and different karyotype formulas. This indicates that karyomorph B originated from karyomorph A (with only acrocentric chromosomes), where centric fusions were the most probable mechanism behind the presence of six metacentric chromosomes in karyomorph B (Fig. 3c). However, another scenario can also not be ruled out, in which fission-type rearrangements would have originated the additional 12 acrocentric chromosomes in karyomorph A. Once the majority of chromosomal rearrangements involve heterochromatic regions, especially in fish species [26, 27], this explains the centromeric rearrangements in *M. albus*. The co-localization of CMA_3_ positive heterochromatin with the NOR loci also occurs in other synbranchids [25, 28], probably because of local changes in base composition (increase in GC content) due to the so-called GC-biased gene conversion that involves rDNA in many vertebrates, including ray-finned fishes [29].

Although the 18S and 5S sites are located in an acrocentric pair in karyomorph A and in a metacentric pair in karyomorph B, it is possible to infer that both pairs are related in both karyomorphs. As karyomorph B contains additional 18S rDNA signals in the telomeric region in pair no. 3, it is likely that a pericentric inversion has divided the pericentromeric 18S rDNA loci into two parts and has transposed one of them near the telomere. Centric fusions would be the most suitable rearrangements to illustrate this scenario.

The differential distribution of the microsatellite motifs (CA)_15_ and (TA)_15_ between the two karyomorphs reinforces the interpretation that they represent, in fact, different species. A genome-wide analysis found that microsatellites have a repeat- and chromosome-biased distribution in *M. albus* from China, mainly located in noncoding regions (98,602, 99%) [30]. Differences both in the abundance and in the chromosomal location of several microsatellite motifs have been reported among closely related fish species also involved in recent speciation events [31, 32]. Similar to microsatellites, the *Rex1* sequences are also resolutive markers for comparative genomic studies, as was already shown for several Asian fish species [19, 30, 31]. In the present study, the interspersed distribution of the retroelement *Rex1* contrasts with that reported by [19].

Inter- and/or intrapopulation diversity has also been found in the karyotypes of other Synbranchidae species. For example, *Ophisternon aenigmaticum* and *Synbranchus marmoratus* possess high karyotype variability, with several karyomorphs described among their populations; they are also considered species complexes, where independent and bidirectional rearrangements, such as fusion and fission events, were responsible for the distinct 2n and karyotypic characteristics observed (Table 1). Biological, physiological and/or reproductive characteristics of Synbranchidae fishes may facilitate the intra- and interspecific karyotype variability observed among species and populations, especially in *Monopterus* and *Synbranchus*. These species tolerate a wide range of water oxygen levels, being able to obtain up to 25% of air oxygen by the cutaneous surface, allowing them to survive up to nine months in a drying burrow [33]. They also form small populations, and although they prefer freshwater habitats, these fishes tolerate brackish and saline conditions [1]. On the other hand, as many individuals can be isolated in small lakes during dry years, the capacity of sex reversal observed in synbranchids could contribute to the viability of such populations, increasing the probability of the fixation of genetic differences and speciation processes [25, 28].

## Conclusions

In summary, the present scenario points to the occurrence of a chromosomal speciation scenario, in which fusions and inversions had a prominent role in the diversification of two distinct karyomorphs that differ with respect to diploid number, chromosome features and repetitive DNA distributions. However, this does not necessarily mean that a sympatric speciation is the only viable alternative, since such karyomorphs could have been established in allopatry, where a secondary join between them originated the present distribution. The karyotypic features highlight the biodiversity of *M. albus* and justify a taxonomic revision, since this nominal species may actually constitute a species complex.

## Methods

### Individuals examined

Two hundred specimens of *M. albus* were collected in 22 localities from distinct Thai regions (Fig. 2 and Table 2). The specimens were caught using traps, and after capture, the animals were placed in sealed plastic bags containing oxygen and were transported to the research station. The specimens were deposited in the fish collection of the Cytogenetic Laboratory, Department of Biology, Faculty of Science, Khon Kaen University. All the experiments followed ethical protocols, and anesthesia with clove oil was used prior to sacrificing the animals to minimize suffering. The fishes were then immersed in an ice-slurry to achieve death by hypothermia. The process was approved by the Animal Ethics Committee of Khon Kaen University based on the Ethics of Animal Experimentation of the National Research Council of Thailand AEKKU23/2558.

### Chromosome preparation and C-banding, ag- and CMA_3_ staining

Mitotic chromosomes were obtained from the cell suspensions of the anterior kidney, using the conventional air-drying method [34]. Conventional staining was done using 5% Giemsa solution in phosphate buffer, pH 6.8, for 10 min. Chromosomes were analyzed after silver nitrate staining [35] in order to visualize the nucleolar organizing regions (Ag-NORs), and C-banding was also employed to detect the C-positive heterochromatin [36]. GC-specific fluorochrome chromomycin A_3_ (CMA_3_) was carried out following the method of Amemiya and Gold [37] to detect CG-rich regions on the chromosomes.

### Preparation of FISH probes derived from repetitive sequences

Two tandemly arrayed rDNA sequences isolated from the genome of an Erythrinidae fish species, *Hoplias malabaricus*, were used as probes, as described in details in [38, 39]. The 5S and 18S rDNA probes were labeled with Spectrum Green dUTP and Spectrum Orange dUTP, respectively, using nick translation according to the manufacturer’s recommendations (Roche, Mannheim, Germany).

The microsatellites (CA)_15_, (GA)_15_,(GC)_15_, (TA)_15_, (CAA)_10_, (CAC)_10_, (CAG)_10_, (CAT)_10_, (CGG)_10_, (GAA)_10_ and (GAG)_10_ were synthesized according to Kubat et al. [40]. During synthesis by Sigma (St. Louis, MO, USA), these sequences were directly labeled with Cy_3_ at the 5’terminus. The retrotransposable element *Rex1* sequence was prepared by PCR, using primers described in Volff et al. [41]. The *Rex1* probe was directly labeled with Spectrum Orange dUTP by nick translation, according to the manufacturer’s recommendations (Roche, Mannheim, Germany).

### Detection of repetitive DNA sequences by FISH

All FISH experiments with repetitive DNA probes were essentially carried out according to the protocol described in Yano et al. [42]. The first post-hybridization wash was performed with 2x SSC for 5 min at 42 °C, and a final wash was performed at room temperature in 1x SSC for 5 min. Finally, the slides were counterstained with DAPI and mounted in an antifade solution (Vectashield from Vector Laboratories).

### Microscopic analysis and image processing

At least 30 metaphase spreads per individual were analyzed to confirm the 2n, karyotype structure and FISH results. Images were captured using an Olympus BX50 microscope (Olympus Corporation, Ishikawa, Japan) with Cool SNAP, and the images were processed using Image Pro Plus 4.1 software (Media Cybernetics, Silver Spring, MD, USA). Chromosomes were classified as acrocentric (a) or metacentric (m), according to their arm ratios [43].

## Abbreviations

2n: Diploid chromosome number
a: Acrocentric chromosome
CMA_3_: Chromomycin A_3_
DAPI: 4′,6-diamidino-2-phenylindole
dUTP: 2′-Deoxyuridine-5′-Triphosphate
FISH: Fluorescence in situ hybridization
FN: Fundamental number
m: Metacentric chromosome
NOR: Nucleolar organizer region
NTS: Non-transcribed spacer
PCR: Polymerase chain reaction
rDNA: ribosomal DNA
sm: Submetacentric chromosome
st: Subtelocentric chromosome
